# Supplementary material for: Plasma Lipidomics Analysis Reveals the Potential Role of Lysophosphatidylcholines in Abdominal Aortic Aneurysm Progression and Formation
Source: Int J Mol Sci. 2023 Jun 16;24(12):10253. doi: 10.3390/ijms241210253 (PMC10299432; doi:10.3390/ijms241210253)
Supplement: Supplementary file 1 [file ijms-24-10253-s001.zip › ijms-2423300-supplementary.pdf]

Figure legends

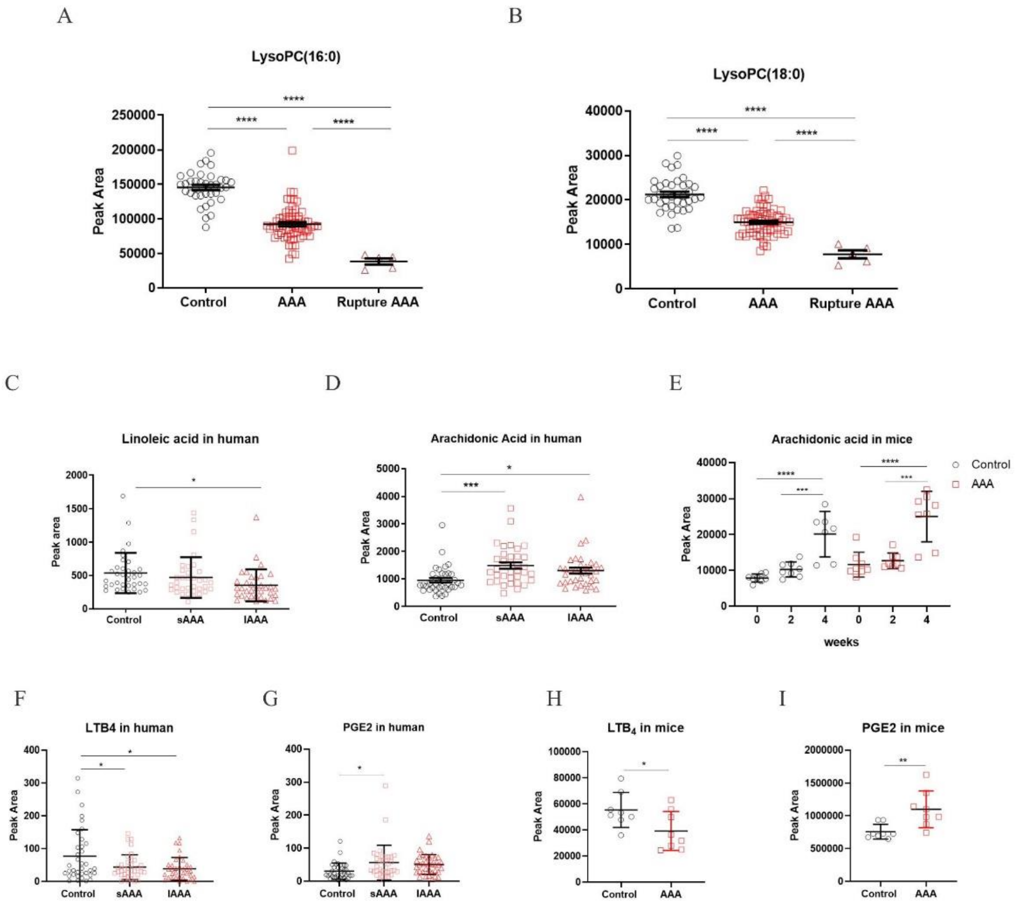

Supplementary Figure S1. Scatter diagram for lipids in human and mice. (**A**) LysoPC (16:0) in controls (n = 36), AAA (n = 65) and rupture AAA (n = 5). (**B**) LysoPC (18:0) in controls (n = 36), AAA (n = 65) and rupture AAA (n = 5). (**C**) Arachidonic acid in controls (n = 36), sAAA (n = 35) and lAAA (n = 35). (**D**) Arachidonic acid change in mice at 0, 2, 4 weeks in controls (n = 8) and AAA (n = 8) mice. (**E**) Linoleic acid in controls (n = 36), sAAA (n = 35) and lAAA (n = 35). (**F**,**G**). Leukotrienes B4 (LTB4) and Prostaglandins E2 (PGE2) in controls (n = 36), sAAA (n = 35) and lAAA (n = 35). (**H**,**I**) Leukotrienes B4 (LTB4) and Prostaglandins E2 (PGE2) in mice at 4 weeks in controls (n = 8) and AAA (n = 8) mice. *p < 0.05, ***p < 0.01,***p < 0.001, ****p < 0.0001.
